# Hospital and Institutional News

**Published:** 1913-09-06

**Authors:** 


### HOSPITAL AND INSTITUTIONAL NEWS.

### DUBLIN RIOTS AND JERVIS STREET HOSPITAL.

The inevitable results of the riots in Dublin, which occurred more or less throughout the past week-end, have been to crowd the Jervis Street Infirmary with patients, and to excite the bitterest feelings between a section of the public and the police. With the latter point we have no province to deal, but the importance of the former can be judged by the fact that on Sunday last no fewer than 95 civilians were treated at Jervis Street Hospital. By Tuesday morning last the casualties amounted to 433, and were said to be distributed among the hospitals as follows: To the Jervis Street Infirmary, 320; to Sir Patrick Dun's Hospital, 63; and to the Mercer's Hospital, 50. English readers may remember that the Jervis Street Infirmary, which is the oldest of the three institutions, contains 100 beds, the Mercer's Hospital 77, and Sir Patrick Dun's 108. The bulk of the work fell upon the Jervis Street institution.

### OUR EDUCATIONAL NUMBER.

Owing to the exceptional pressure on our space we have been compelled to hold over some articles we had hoped to include in this issue. One, which concerned the London School of Tropical Medicine, specially illustrated, will probably appear next week. The monthly "Institutional Library" has also been crowded out, but will appear next week, with several reviews of important hospital books.

### THE THREAT TO CLOSE HOSPITAL BEDS.

Dealing with our criticisms on cathedral cities, the *Eastern Weekly Press* regretfully remarks that Norwich is travelling on the same road as Worcester in the financial sense, but has not yet reached the fatal edge. The writer then goes on to assume that those people who threaten the public with the closure of a ward are speaking with good cause, whereas, of course, in fact hospital knowledge demonstrates that such threats are idle in the sense that they can have no genuine force. The reason is that when a hospital is in financial distress the closing of a ward, or wards, will not substantially help the institution's finances, for such a step merely tends to increase considerably the cost per bed occupied. It does little relatively to reduce the expenditure rendered necessary by the maintenance of a hospital capable of satisfying all the needs of the sick resident within the district which it serves.

### THE YORK COUNTY HOSPITAL INQUIRY.

Similarly, the *Yorkshire Gazette*, discussing our criticism on hospital management in cathedral cities, remarks that we have "overlooked the fact that many of the 'affairs' which were revealed during the York County Hospital inquiry had been remedied before the inquiry took place." We beg to assure our contemporary that we have not overlooked this fact, but that, on the contrary, as anyone who reads the verbatim report of the inquiry must realise, the so-called remedies were mainly palliatives to disarm criticism. They not only lacked thoroughness but they afforded no evidence of a desire to get to the root of the mischief and to remove the actual cause of the abuses proved to have been tolerated in this hospital. The so-called remedies fail to be effective, for they do not touch the high officials and managing authorities responsible for an administration under which abuses had been permitted to prevail in some cases for a lengthened period. The whole system on which the affairs of the York County Hospital was shown to be administered, as the inquiry demonstrated, was lamentably faulty. Until this faulty administration is abolished in favour of a modern, up-to-date system, and those responsible for the former are changed, there can be no hope of real efficiency in the modern-hospital sense at the York County Hospital.

### ISOLATION HOSPITAL TABOOED BY PATIENTS.

Owing to an epidemic of scarlet fever which has broken out at Greenford in Middlesex, the District Council has improvised an isolation hospital, which, however, the patients, with one amenable exception, refused to enter. The case being desperate, a magistrate's order was issued "requiring" patients, where considered necessary, to be removed. The order, however, has been defied, and it is stated that a nurse who tried to persuade the patients to be removed was obstructed and indeed suffered some personal affront. Defeated of its immediate object, we understand that the Greenford District Council is instituting proceedings against the contumacious persons who "disobeyed or obstructed the carrying out of the order." There is, of course, such a well-known thing, even outside theological controversy, as invincible ignorance, but we can hardly believe that this alone can account for such an unwonted display of resistance among our countrymen, who usually deserve the popular description of the Englishman as a law-abiding person. Panic measures—and an improvised isolation hospital is, in no necessary invidious sense, among these—are not always as well thought out as they might be. So until the nature of the objections of the patients be disclosed, which should be the most interesting point of the proceedings to be undertaken, we shall keep an open mind on the rights and wrongs of this exceptional situation.

### MECHANICAL RESTRAINT OF MENTAL PATIENTS.

New regulations to govern the use of instruments and appliances for the mechanical restraint of mental patients have just been circulated by the Commissioners in Lunacy. They are to come into force on October 1, 1913, and are to take the place of the regulations of April 17, 1895. The new regulations are more precise and detailed than the old and they make provision for the sanction of the use of appliances for restraint other than those actually prescribed. A straight jacket may be used different from that described if it has been approved under the seal of the Commissioners and a sample bearing the seal is kept at the institution for inspection. Also, if, in the opinion of the medical man in charge of the lunatic, some mechanical means of bodily restraint other than those permitted is necessary in a particular case where the circumstances are exceptional, such means may be used with the previous sanction of the Commissioners for such periods as they may authorise. Gloves are to be considered as mechanical means of restraint only if so fastened that they cannot be removed by the wearers. If the continuous bath is employed the use of a cover to it will no longer be deemed to be restraint if the aperture in it for the patient's head is large enough for his body to pass through. Trays or rails fastened to the front of chairs used by idiot children, cripples, or aged infirm adults to prevent their falling out are not to be considered restraint provided that in the case of adults it is within the patient's power to undo the fastening. The greater elasticity of the new regulations will be welcomed by medical men having charge of mental patients, whilst the safeguards introduced are such as should prevent any abuse.

### HARKING BACK TO CONTRACT PRACTICE.

The medical treatment of the uninsured is now seen to be a problem which the Insurance Act is also bringing into prominence. The Carnarvon Oddfellows have written to Mr. Lloyd George complaining that while before the Act came into force a doctor's services could be had for a capitation fee of 4s. a member annually, now the medical men refuse to undertake contract work at a lower rate than that paid by the Government for the treatment of the insured. The aged and non-insured members of the Carnarvon Oddfellows are thereby represented as having a grievance. We suspect that these members, for whom we have the sincerest sympathy and respect, are really but the stalking-horse of the actuaries behind this society. Their interests must, of course, be safeguarded, but why should the insurance society seek to provide for them out of the doctor's pockets? It is admitted on all hands that the rates of contract practice before the Insurance Act was passed were sweating rates. The doctors again and again have pointed out, when arguing in defence of the capitation fees which they succeeded in securing under the Insurance Act, that even these rates were only acceptable because they were paid by the mass of the insured, a proportion of whom alone might be expected to require medical attendance. That being so, it is surely unreasonable that the doctors should be asked to go back to the old sweating terms in respect of the very class of patient which is most exacting on the medical profession. The interests of the aged uninsured members of friendly societies must be safeguarded, but the cost must not be thrown upon the doctors. The societies exist to bear it, and the reason that they exist to bear it is because they possess large revenues, which no one pretends it is either possible or right for the medical profession to own or to hold. In the face of these facts Mr. Lloyd George's answer to the Carnarvon Oddfellows that as "under the Insurance Act he (the doctor) is able to earn a larger remuneration than before, in respect of insured members of the society . . . he could continue his treatment of the aged uninsured as in the past," is seen to be the hollowest pretence of political special pleading. Neither the uninsured, the doctors, nor the public will be deceived by it.

### PANEL DOCTORS AND MALINGERING.

A member of the Brighton and Sussex Mutual Provident Approved Society was summoned last week on the charge of having knowingly made a false declaration to obtain half a sovereign. The case has special interest from the fact that the attitude of the medical profession, or rather of that section which is on the panel, was called in question. The point as regards the panel doctor may be expressed by a remark of one of the Brighton magistrates, who said that they had direct evidence that the defendant was at work when two of the certificates (purporting to represent incapacity) were signed. Dr. Basil Jackson, in reply, said: "A panel doctor has no chance, when a patient is a hospital patient, of doing more than I did. A patient may come to the doctor and say the hospital doctor has ordered him to lay up for a week, or twenty, and you cannot examine him because he is another doctor's patient. If you do not sign the certificate the poor, wretched patient does not get his money. What is a panel doctor to do?" The presiding magistrate sentenced the defendant to six weeks' imprisonment with hard labour, and said: "The magistrates regret that such laxity should have occurred. We feel that this contributed to the offence, and we hope that greater care will be exercised in the future." Dr. Jackson's excuse will not bear examination.

### THE MEDICAL REFEREE AS A CURE FOR MALINGERING.

The approved societies are so impatient to end the losses which they declare to be now affecting them through malingering that some have decided not to wait till the recently instituted Departmental Committee's inquiry be completed, but in the meantime to make provisional arrangements to protect themselves. It seems that the more active societies may take advantage of a circular issued by the Insurance Commissioners, and secure the services of medical referees. Any society and doctor may make arrangements of this kind, and joint schemes covering several societies may be concluded by an Insurance Committee on their behalf. Such joint arrangements, however, throw on the societies all administrative expenses and doctors' fees, and the position of the Committee is in regard to them simply that of an agent. The Insurance Commissioners point out in their circular that any type of arrangement must be both provisional and temporary, since no vested interest may be created until the Committee has issued its report. A temporary financial adjustment is authorised in cases where the amount at present available under existing regulations for expenditure connected with referees is small. The society in any case is to be responsible for the cost. The medical referee, we need hardly say, can prove no panacea for the cure of malingering, but, if properly paid, will be free from the overwork and allied temptations which tend to make the panel doctor an uncritical granter of certificates.

### THE LITERATURE OF HOSPITALS.

Some thirty years ago, when we began to publish The Hospital as a weekly newspaper, many critics expressed the view that there was no opening for such a journal. One dear friend, now dead, who was a great authority on hospitals in the United States, bluntly wrote his congratulations with the warning and forecast that The Hospital might continue to appear for one whole year because of the energy behind it, and then facts and natural causes would end its existence. The United States of America have had since the days when the American Hospital Association was established, and as a result of that most excellent new departure, a monthly journal, often containing information of value and facts of importance, entitled *The International Hospital Record.* Recently this journal has developed into a weekly digest of information pertaining to hospital and allied institutional work and nursing. The new form is not convenient to handle, owing to the large size of the pages—imperial royal, we should call it—and the fact that some numbers contain only fifteen pages, including advertisements. We merely mention the fact because we hope our old and valued *confrère* will put the matter right, and so provide for many years of increasingly useful development in its new form. The size in many ways was an advantage in the old form, but in its present form it has the disadvantage we have indicated.

### DISTRICT MEDICAL OFFICER ASKED TO RESIGN.

At a meeting of the Poplar Board of Guardians last week, the decision of the Local Government Board with reference to the inquiry recently instituted into certain allegations against Dr. J. D. Hendry, district medical officer, was made known. As we noted at the time of the inquiry, which was held by Mr. Oxley, a Local Government Board inspector, Dr. Hendry was charged with having offered money, on behalf of a lady recently appointed to a whole-time dispenser's post, to two Guardians, by way of recognition of their having given their votes in favour of the lady's candidature. The Local Government Board have asked Dr. Hendry to resign, giving their reasons for this course in a letter from which the following is perhaps the most significant passage: "Mr. Oxley has now submitted to the Board his report, together with the evidence of the witnesses taken at the inquiry. Mr. Oxley finds that the charges made against you by Messrs. Adams and Munnings are substantially true, and the Board, after careful consideration of the matter, are of opinion that this conclusion is supported by the evidence. In the circumstances the Board regret that they have no alternative but to call upon you to tender your resignation as district medical officer, and I am to request that you will place your resignation in the hands of the Guardians forthwith." The Guardians, who also received a copy of the letter, without comment, referred it to the general purposes committee. The need for this decision is very regrettable, but on the result of the inquiry it was no surprise.

### INSURANCE IN GERMANY AND ENGLAND.

We have received an advance copy of a paper on this subject prepared by Dr. D. J. Mackintosh to be read at the Conference of the American Hospital Association at Boston, Mass., on the 28th ult. This paper is a summary of the principal provisions and bearings of the National Insurance Act, 1911, with an explanation of sanatorium benefit and hospital treatment, so far as it deals with National Insurance in Germany, together with a summary of Dr. Gibbon's book, "Medical Benefit in Germany and Denmark." The figures, tables, and information contained on page 178, for instance, and in other sections of the book are given, but without any indication that they are quoted, the only reference to Dr. Gibbon's work being on the last page (31) of Dr. Mackintosh's paper in which he says: "I would like to refer to the valuable information I have obtained from the work of Mr. I. G. Gibbon." The paper is a great disappointment to us because we had hoped to find much original information seeing the position Dr. Mackintosh has deservedly made for himself in this country as a hospital expert.

### SHOULD POLICE-WATCHING OF SUICIDES IN HOSPITALS BE ABOLISHED?

According to a recent announcement the City Police were successful in 1912 in preventing thirty suicides, and during the same period the number of suicides committed within the technical precincts of the City was fifteen. The reading of these statistics inevitably excites reflections upon the attitude of the law to the subject of suicide, and upon the way in which this attitude affects the position of hospitals, the aid of which in unsuccessful attempts at self-destruction is, of course, so often invoked. The humour which lies at the heart of every really grave matter is nowhere better seen than here. "The law of England," we read in the sixth edition of Taylor's "Principles and Practice of Medical Jurisprudence," edited by Dr. F. J. Smith, "treats suicide as a felony; those who have attempted and failed in the perpetration are held to be sane and responsible agents, unless there should be clear evidence of their (intellectual) insanity from other circumstances." In other words, the action of the law in punishing unsuccessful attempts at suicide, presumably inflicted on deterrent grounds, really uses all the force of society to ensure that the attempt shall be successful. Perhaps this may be true of punishment in general, for a successful suicide is on a par with an undetected felony; that is to say, it allows the criminal to evade the reach of the law. Turning, however, to the position in which hospitals are placed by the law in relation to cases of attempted suicide, Dr. Smith quotes an instance in which "the police ordered that such cases should not be admitted to a particular general hospital because the hospital authorities could not or would not pay for police-watching. In the editor's opinion (Dr. Smith continues) such police-watching is detrimental to everybody and a nuisance to the hospital." Not everyone will endorse this opinion. Hospital secretaries have told us that policemen in wards are not invariably a nuisance; that they relieve the staff of an unpleasant responsibility; and that in many cases the policeman is only too anxious to relieve the tedium of his empty hours by assisting in certain humble but useful ways in the work of the ward where he is stationed. We invite the experience of our readers on this point.

### REFORMING THE LAW BY PRIVATE ENTERPRISE.

While on the subject of suicide we cannot forbear to reproduce certain sections of a very remarkable letter which was read by the Coroner at an inquest at Barnet on the body of an aged man which was found in a ditch with a bottle nearly full labelled "Chloroform." The evidence showed that the deceased was a German subject, entitled to the German degree of Ph.D., who was a teacher of foreign languages and had been in England for some forty-five years. The letter, abbreviated, read:

Dear Sir (meaning the Coroner),—I am sorry to give you the trouble of coming to see me. I took three-shillingsworth of laudanum, and if that has not the wished-for effect I shall be very disappointed. I am not out of my senses—hale in body and mind, eighty-two years old. Have never been ill for the last fifty years, but, I am sorry to say, without the necessary means of living. I have always tried to keep out of debt, and have succeeded in that task, and given what help I could to my fellow-men; and in order that this death of mine should and might be of some advantage to my fellow-men, I shall be obliged if you can hand my body over to some school of anatomy for dissecting in order to find out the reason why I have reached so high and vigorous old age, and thus do a favour to the medical profession. Hoping you will excuse my writing to you, with great respect I am, dear Sir, yours respectfully, A. H.

The Coroner is reported to have remarked that the old gentleman was a bit of a wag, and the jury returned a verdict that death was due to chloroform poisoning accelerated by cold and exposure, and added that the chloroform was "self-administered with a view to self-destruction." By the Interments (Felo de Se) Act, 1882, Taylor remarks that the Coroner is to direct the remains to be interred in a churchyard or other burial ground of the parish, subject to the provisions of the Burial Laws Amendment Act, 1882. "It was hoped," adds the writer, "that this enactment would do away with many absurd verdicts of 'temporary insanity,' but it has failed to do this." What Parliament was unable to do by amended legislation "A. H." has accomplished in his own case. In spite of the initials to the letter the name of the deceased was given at the inquest as Julius Reusch.

### THE LONDON HOSPITAL AND THE ALMONER DIFFICULTY.

The quarterly report of the house committee of this hospital records that the institution has received in the past most valuable assistance from the Hospital Saturday Fund, and that it was with great regret that the committee had to disagree with a resolution of the Fund with regard to the inquiries made by almoners. The Hospital Saturday Fund asked the London and other hospitals that, when a patient was sent to the hospital furnished with a letter of recommendation from the Fund, the hospital should accept and honour such recommendations without further inquiry. The committee felt, however, that the almoners were appointed to protect the Hospital Saturday Fund, as a subscriber, just as they were to protect all other subscribers, and therefore decided, as a matter of principle, to refuse to agree to such conditions. We understand every large hospital in London has done the same.

### CLAUSE 12 NO BENEFIT TO THE VOLUNTARY HOSPITAL.

With regard to the Insurance Act, the London Hospital has devised a scheme whereby when insured patients treated in the hospital have no dependants the friendly societies concerned should make a contribution to the hospital towards the expense of the patient's treatment. The scheme has met with no success, however, all friendly societies, with one exception, refusing to make any grant to the hospital, although the Act gave them powers to do so. The house committee points out that the latest amendment of the Act empowers the friendly societies to give to patients the sickness benefit after they leave the hospital, even if they have no dependants. It seems useless, it is felt, to spend further time and labour in trying to obtain these contributions, but it is not thought a fair way of treating the hospital which has done the work and borne the expense.

### THE MEANING OF RESEARCH IN MEDICAL EDUCATION.

Among the many criticisms of the Report of the Royal Commission on London University, none is more interesting to the hospital world than that which Dr. Charles Mercier has embodied in a letter to the Educational Supplement of *The Times*. He insists on a proper understanding of the meaning of Research in relation to medical education. After pointing out that according to the Commissioners "the medical undergraduate of the University should be brought up in an atmosphere of 'research,'" he declines to have this word limited to "some recondite method of finding out what is unknown," and argues very pertinently that the whole time which the student spends in the wards is spent in research into the particular things which he will be called upon to investigate in after-life. To research, Dr. Mercier emphasises, means no more than to search diligently, and he argues that the best way in which to teach students to do this is as much the province of clinical instruction as of specialised laboratory work. There is an undoubted danger, just now, of this side of the question being forgotten, and Dr. Mercier is to be congratulated for bringing the straying attention of the disputants sharply to the halt.

